# Preventive care for adults with Down syndrome in Connecticut: a mixed-methods study

**DOI:** 10.1017/S1463423626101170

**Published:** 2026-03-27

**Authors:** Andrew Brandser, Lucas Cordova, Emily R. Shearier, Ingrid Philibert, Traci Marquis-Eydman

**Affiliations:** 1 Family Medicine, Quinnipiac University Frank H Netter MD School of Medicine, North Haven, USA; 2 Quinnipiac University Frank H Netter MD School of Medicine, USA; 3 Hartford Healthcare, USA

**Keywords:** Adults, Down syndrome, preventive care, primary care

## Abstract

**Aim:**

This study examined gaps in adherence to preventive care recommendations for adults with Down Syndrome (DS) in Connecticut and explored the underlying factors collecting caregiver and primary care physician (PCP) perspectives.

**Background:**

Primary healthcare plays a vital role in preventing health issues. Despite well-defined clinical guidelines for adults with DS, studies show gaps in preventive care delivery for this population.

**Methods:**

A mixed-methods study included chart reviews, a focus group and a survey of PCPs. Chart reviews examined records of adults with DS who received care between January 1, 2017, and December 31, 2022, for adherence to recommended preventive services. The focus group explored caregivers’ experiences with preventive care, and the survey assessed PCPs’ knowledge of prevention needs for adults with DS.

**Findings:**

Chart reviews of 241 adults with DS found low adherence to preventive care guidelines. Only 2.1% met the wellness visit benchmark, and 30.7% met the thyroid test benchmark. Themes from the caregiver focus group included challenges accessing care, clinicians’ lack of DS-specific knowledge and difficulties maintaining health and wellness outside the office setting. Of 81 PCPs surveyed, most reported feeling inadequately prepared to care for adults with DS. Only 27% reported relevant training, and 53% were unaware of annual thyroid function test recommendations.

**Results and Conclusions:**

The study reveal gaps in preventive care for adults with DS and underlying reasons from a caregiver and provider perspective. Further analysis of care for adults with DS and targeted interventions will contribute to improved preventive care for this population.

## Introduction

Adults with Down syndrome (DS) face unique healthcare challenges that require reliable preventive care and specialized medical attention. Despite affecting approximately 1 in 700 live births in the United States, significant gaps exist in the delivery of preventive healthcare services to this population. While established guidelines recommend specific preventive measures such as annual wellness exams and thyroid function testing, adherence to these guidelines remains inconsistent and poorly documented. Access to primary care and the effectiveness of preventive care have been shown to have significant positive effect on health outcomes (Shi, 2012).

Access to primary care has an even greater impact on individuals with chronic health conditions and those who utilize healthcare more frequently (Brundisini *et al.*, 2013; Dassah *et al.*, 2018). For individuals with disabilities, there are additional barriers such as adequate training of primary care physicians (PCPs) and patients and/or their caregiver having insufficient resources to manage the disability (Dassah *et al.*, 2018).

As the most common genetic cause of intellectual and developmental disability, DS presents unique challenges for primary care delivery. In the primary care setting, the American Academy of Family Physicians (AAFP) recommends additional annual measures beyond standard screenings to assess for increased risks of hypothyroidism, obesity, and depression in adults with DS (Smith, 2001). Studies have shown that adults with DS experience challenges with ensuring regular primary care visits as well as with the services provided during these visits (Jensen *et al.*, 2013; 2021a; 2021b).

From the perspective of caregivers of children with DS, some shortcomings appear to result from the inability of the PCP to fully understand what support is needed (Skelton *et al.*, 2021). Studies have also reported care givers’ lack of awareness of available resources or difficulty accessing them, especially for rural and lower-income communities (King *et al*., 2022). No comparable caregiver perspectives exist for adults with DS. The relative lack of knowledge about specific guidelines for the care of adults with DS contributes to a reduced frequency of planned healthcare visits and an associated increase in the frequency of unplanned visits (Ahlström *et al.*, 2020).

While the complexities of primary care for adults with DS have been described, few studies have analyzed this using objective data and the perspectives of caregivers and primary care providers. Our study sought to identify gaps in adherence to preventive care services for Connecticut adults with DS receiving care at one health system and explore causative factors using caregiver and provider perspectives. To facilitate this, we (1) conducted chart reviews to examine whether PCPs met established guidelines for preventive care for adults with DS; (2) explored caregiver perceptions of the quality of preventive healthcare for adults with DS; and (3) collected data on PCPs’ self-reported knowledge about and comfort with providing preventive care for adults with DS. Our aim was to identify existing disparities for this population and to suggest pragmatic solutions.

## Methods

### Retrospective chart review

Our chart review focused on patient visits to Hartford HealthCare (HHC) primary care sites over a six-year timeframe between January 1, 2017, and December 31, 2022.

The chart review population included patients aged 18–60 with DS (CID-9-CM: 758.0; ICD-10-CM: Q90.9) who sought care in the HHC system during the survey period and had an HHC PCP. Patient residence was classified as rural versus urban using the *Rural-Urban Commuting Area Codes* (U.S. Department of Agriculture Economic Research Service, 2020). Minimum age was chosen as the date of entry into adulthood; the maximum age took into account the average lifespan of individuals with DS (Lulita *et al.*, 2022). Payer type reflects the patient’s primary insurance.

The chart review data were extracted from Hartford HealthCare Epic database by an institutional data scientist using the inclusion criteria and national preventive care recommendations for patients with DS. The RUCA codes were obtained from the reference website as an Excel file.

The preventive care measures in our analysis were based on the American Academy of Family Physicians (AAFP) preventive care guidelines for adults with DS and prior studies (Smith 2001; Jensen *et al.*, 2013; 2021a; 2021b). The primary care measures included (1) completion of an annual wellness exam and (2) collection of a thyroid function test (TFT). The TFTs included orders for TSH, T4, or free T4. The annual wellness exam codes and thyroid function labs were identified via the ICD-10 and Current Procedural Terminology (CPT) codes. Meeting AAFP preventive care guidelines specific to adults with DS during the 6-year study period was measured as an annual wellness visit and annual thyroid function test (Smith, 2001). Patients were considered to meet these guidelines if they met the benchmarks of 6 total thyroid tests in 6 years, an average time of one year or less between thyroid tests, 6 wellness visits in 6 years, and an average time of one year or less between wellness visits. The specific codes are as follows: *Well Child Examinations* (patients ≥18 may still see their pediatrician): ICD-9 V20.2; ICD-10 Z00.129; CPT 99381-99384, 99391-99394, *Well Adult Examinations: I*CD-9 V70.0, V72.3,.2; ICD-10 Z00.0V76; CPT 99385-99387, HCPS G0402, S0610, S0612, S0613, and *Thyroid Function Tests*: CPT 80091, 80092, 84436, 84439, 84443, 84479.

Additional data collected included dates of emergency department (ED) and urgent care (UC) visits. These were grouped as ‘unplanned visits’. Usage of ‘unplanned visits’ assessed the frequency of utilizing an ambulatory services other than a primary care setting, which is the typical location for receiving preventive care (Shi, 2012).

### Focus group

Focus group participants were recruited through collaboration with leadership at Special Olympics Connecticut, Hartford HealthCare, and the Down Syndrome Association of Connecticut. Study recruitment materials were distributed electronically and included a flier describing the study and the $25 compensation.

Eligible participants were caregivers for an adult with DS meeting our inclusion criteria. Demographic information on caregivers includes gender, race/ethnicity, and relationship to the adult with DS. For location, the study used the zip code of the adult with DS, because not all caregivers lived with the individual for whom they provide support.

A single focus group was conducted using Zoom video conferencing technology, lasting 90 minutes. All participants had their cameras on; their names were removed during the call to maintain confidentiality.

A trained moderator facilitated the focus group. Focus group questions were taken from the ‘Inclusive Health Needs Assessment Toolkit’ created by Special Olympics, Inc (Special Olympics, Inc, 2022). The toolkit was developed in agreement with the Center for Disease Control and Prevention (CDC) to assist health care providers in identifying the needs of individuals with intellectual and developmental disabilities (IDD) and their families. The focus group questions are provided as supplemental data.

### PCP survey

Physicians surveyed were family medicine and internal medicine PCPs in the HHC system. The 24 survey items also were derived from the ‘Inclusive Health Needs Assessment Toolkit’, with a focus on assessing respondents’ perspectives on preventive care for adults with DS. Survey participants received an email from an HHC liaison containing a link to access the REDCap survey. The email explained the purpose of the survey and provided contact details for a member of the research lead if the participant had any questions before signing the consent. The link was sent twice and a note about the study was included in the Hartford HealthCare newsletter.

### Ethics approval

The study was approved by the Hartford HealthCare Institutional Review Board. The chart review approved on March 7^th^, 2023, received a waiver of informed consent as a retrospective review, protocol number: HHC-2023-0043. Focus group approved on May 10^th^, 2023, participants provided written informed consent and received $25 compensation, protocol number HHC-2023-0075. For the PCP survey approval on February 29^th^, 2024, participants indicated their agreement before accessing the survey questions, protocol number: HHC-2024-0044.

### Data analysis

#### Chart review

Descriptive statistics were used to describe the population and the subpopulations by residence. The comparison for continuous information of urban-rural area subpopulations was examined using the Mann-Whitney *U* test because the normality assumption was not satisfied. A Pearson chi-square test or Fisher’s exact test was used for dichotomous data and a Pearson chi-square with/without a post-hoc test for categorical information. One sample Wilcoxon Signed Rank test was used to examine if the subpopulation reached the benchmark (*p* > 0.05 was considered comparable).

#### Focus group

The focus group transcript generated via Zoom was analyzed using ATLAS.ti software. We chose a thematic analysis of the focus group transcript was chosen because of its demonstrated accountability and reliability in qualitative studies (Jowsey *et al.,* 2021; Raskind *et al.*, 2019).

Inductive coding was used to generate the initial codes, a form of analysis frequently used in previous studies (Raskind *et al.*, 2019). These initial codes were reviewed and refined multiple times, before constructing the themes. The themes were then further grouped into two major themes, each with its own sub-themes. The generated codes and themes were reviewed by a four reviewers. Multiple meetings were held to ensure the accuracy of the generated codes as well as to establish consensus on the developed themes.

Based on qualitative research recommendations to utilize further evaluative criteria beyond achieving saturation, a member-checking approach was used to improve the internal validity of the themes (Birt *et al*., 2016, Tight, 2024). The member-checking process involves asking brief open-ended questions to participants to gauge the degree of agreement and to identify any changes of opinion based on a summary of the identified themes. This additional step was used to ensure an enhanced understanding and confirmation of the accuracy of our interpretation of the focus group data. Member checking was conducted one year after the focus group to provide them with sufficient time for reflection, thereby illustrating the consistency of the ideas highlighted in the focus group over time.

#### PCP survey

Categorical variables were represented as a frequency, using percentages. Continuous variables were evaluated for normality of distribution. Those with normal distributions were presented as mean and standard deviation. Those with non-normal distributions were presented as median with interquartile range.

The primary outcome measures were scores on the Resource Perspective Score (RPS) and the Down Syndrome Knowledge Score (DSKS). Both scoring systems were created by our research team, and the scoring criteria can be found in the supplemental data. The RPS assessed providers’ awareness and perspectives about resources that could aid in preventive care for adults with DS. The possible scores range from 0 to 23, with higher scores indicating higher proficiency. The RPS was calculated from questions 11, 14, 17, 18, and 19 in the survey, which can be found in the supplemental data. The DSKS assesses providers’ knowledge about the care for adults with DS. Scores range from 1 to 20, with higher scores indicating better knowledge. The DSKS was calculated from questions 10, 11, 12, 14, 21, and 22.

### Statistical analysis

All statistical analyses were performed using SPSS version 29 (IBM Corp., Armonk, NY). All statistical tests used a significance threshold of *p* < 0.05.

## Results

### Chart review

The chart review included 241 adults with DS with 29 of them living in a rural residence and 212 living in an urban residence (Table 1). The calculation of the average time between wellness visits and thyroid function tests included participants with only two or more given data points. For the average time between wellness visits, the adjusted sample size was 89 individuals (36.9% of the total individuals), 11 rural (37.9% of the rural subgroup), and 78 urban (36.8% of the urban subgroup). Regarding the average time between thyroid function tests, the adjusted sample size was 140 individuals (57.1% of the total individuals), 20 rural (70% of the rural subgroup), and 120 urban (57.1% of the urban subgroup).

**Table 1.** Patient demographics by total, urban, and rural populations. Benchmarks achieved were defined as six thyroid function tests, six annual wellness visits, an average interval between thyroid tests of ≤1 year, and an average interval between wellness visits of ≤1 year

Demographics	Total (*n* = 241)	Rural (*n* = 29)	Urban (*n* = 212)
**Race,** ***n*** **(%)**			
Black or African American	11 (4.6)	0 (0.0)	11 (5.2)
Other	21 (8.7)	3 (10.3)	18 (8.5)
Patient refused	4 (1.7)	0 (0.0)	4 (1.9)
Unknown	19 (7.9)	1 (3.4)	18 (8.5)
White	186 (77.2)	25 (86.2)	161 (75.9)
**Ethnicity,** ***n*** **(%)**			
Hispanic or Latino	24 (10.0)	3 (10.3)	21 (9.9)
Not Hispanic or Latino	188 (78.0)	23 (79.3)	165 (77.8)
Patient refused	5 (2.1)	0 (0.0)	5 (2.4)
Unknown	24 (10.0)	3 (10.3)	21 (9.9)
**Sex,** ***n*** **(%)**			
Female	106 (44.0)	10 (34.5)	96 (45.3)
**Payers,** ***n*** **(%)**			
Medicare	143 (59.8)	22 (75.9)	121 (57.6)
Medicaid	36 (15.1)	2 (6.9)	34 (16.2)
Private insurance	60 (25.1)	5 (17.2)	55 (26.2)
**Type of Visit (median)**			
Emergency room	0 (0–3)	1 (0–6)	0 (0–2)
Urgent care	0 (0–1)	0 (0–0)	0 (0–1)

#### Patient demographics

Table 1 summarizes the demographic characteristics of the study population. The sample included 106 females and 135 males. The median (M) age at the first visit with the PCP for our chart review cohort was 39 years with an interquartile range (IQR) of 28 to 51. Notably, 186 (77%) of the patients were white (the state of Connecticut is 63.9% white (U.S Census Bureau, 2021). Our analysis for payer types found the following: 145 Medicaid (60%), 36 Medicaid (15%), and 60 private insurers (25%).

Across listed demographic characteristics in Table 1, excluding ‘Unplanned Visits’, Pearson Chi-Square Analysis (*p* > 0.05) showed were no statistically significant differences between rural and urban populations utilizing Pearson Chi-Square Analysis, *p* > 0.05.

#### Preventive care adherence

**Figure 1.**
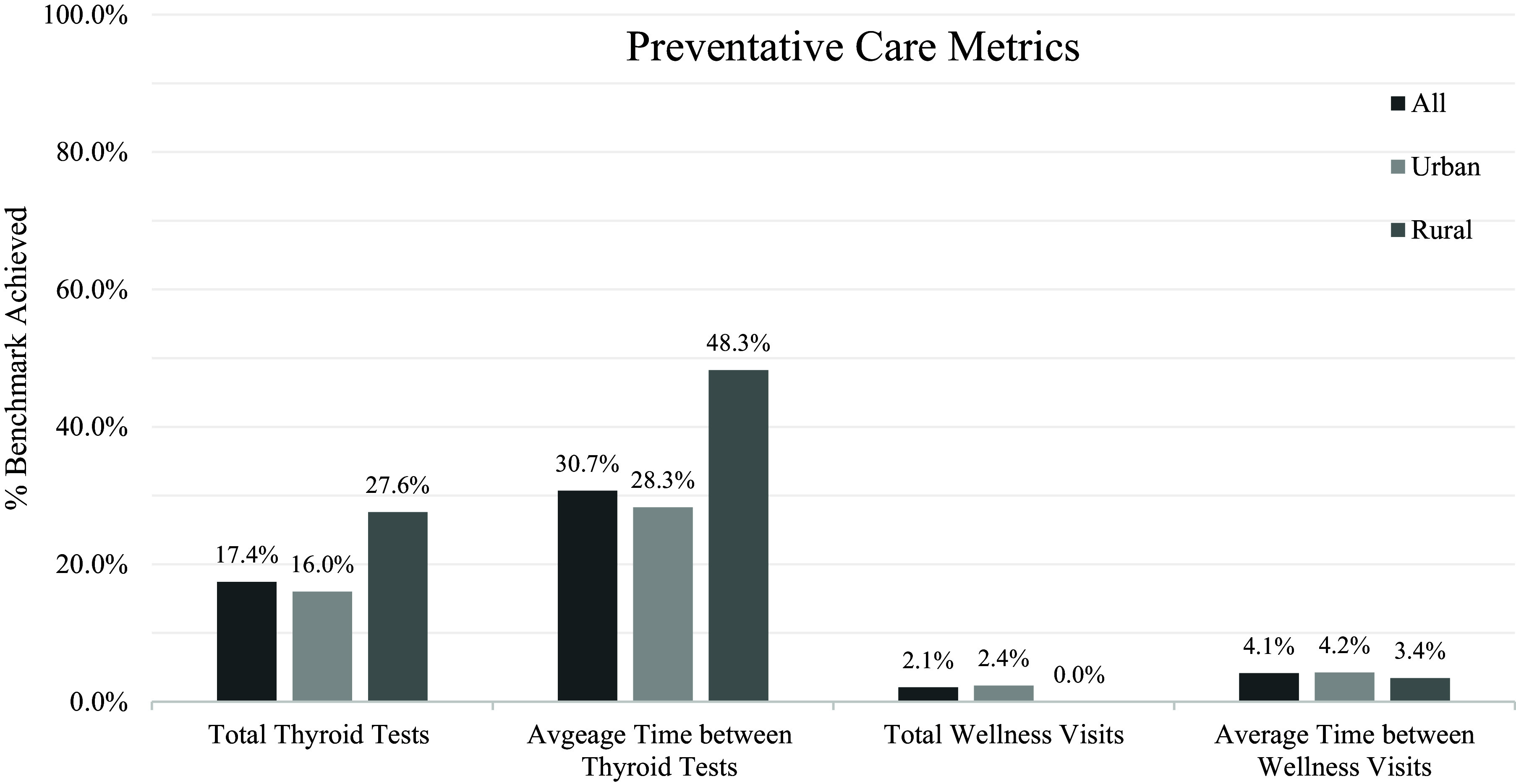
Preventive care utilization by total, urban, and rural populations.

Figure 1 shows the percentage of patients whose care met the guidelines for preventive care for adults with DS. It showed adherence to AAFP preventive care benchmarks was not achieved for the majority of patients in any of the guidelines across all populations. This indicates a low adherence by PCPs to preventive guidelines for adults with DS. The lowest percentage of reaching guideline levels for the patients in our study population was for the recommended number of wellness visits at 2.1% of the patients and the highest was 30.7% for the average time between thyroid tests.

A one-sample Wilcoxon signed rank test revealed statistically significant differences between the total number of wellness visits, *M*: 1, IQR [0–2], *p* < 0.0001, and the total number of thyroid function tests, *M*: 2, IQR [1–4], *p* < 0.0001, compared to the benchmark value of 6. Additionally, the average time between wellness visits for the total population was significantly greater than the benchmark of 1 year, *M*: 1.2 years, IQR [1.0–1.7]. *p* < 0.001. The average time between thyroid function tests for the aggregate population essentially met the benchmark, *M*: 0.97 years, IQR [0.66–1.30], *p* = 0.997.

Our data review did not identify a single individual in the rural patient population who received the recommended number of well visits to a PCP within the study period. However, the rural sub-population had the highest percentage of patients meeting the recommended number of thyroid tests and average time between thyroid tests (27.6% and 48.3%). This difference between the thyroid tests and wellness visits can be explained by the codes for the thyroid function tests being pooled from across the entirety of Hartford HealthCare’s medical system; TFT testing may have been ordered by a specialist such as endocrinology, but we cannot state this with certainty.

#### Emergency room and urgent care utilization

As illustrated in Table 1, rural patients, *M*: 1, IQR [0–6], had a higher number of emergency room visits during the study period than urban patients. *M*: 0, IQR [0–2], *p* = 0.010. Urban patients, *M*: 0, IQR [0–1], had more urgent care visits during the timeframe than rural patients *M*: 0, IQR [0–0], *p* = 0.025. When the emergency room and urgent care visits were combined as ‘unplanned visits’, no significant relationships were found. These secondary results illustrate the need to investigate further the differences in rural and urban sub-populations’ access to primary care.

### Focus group

**Figure 2.**
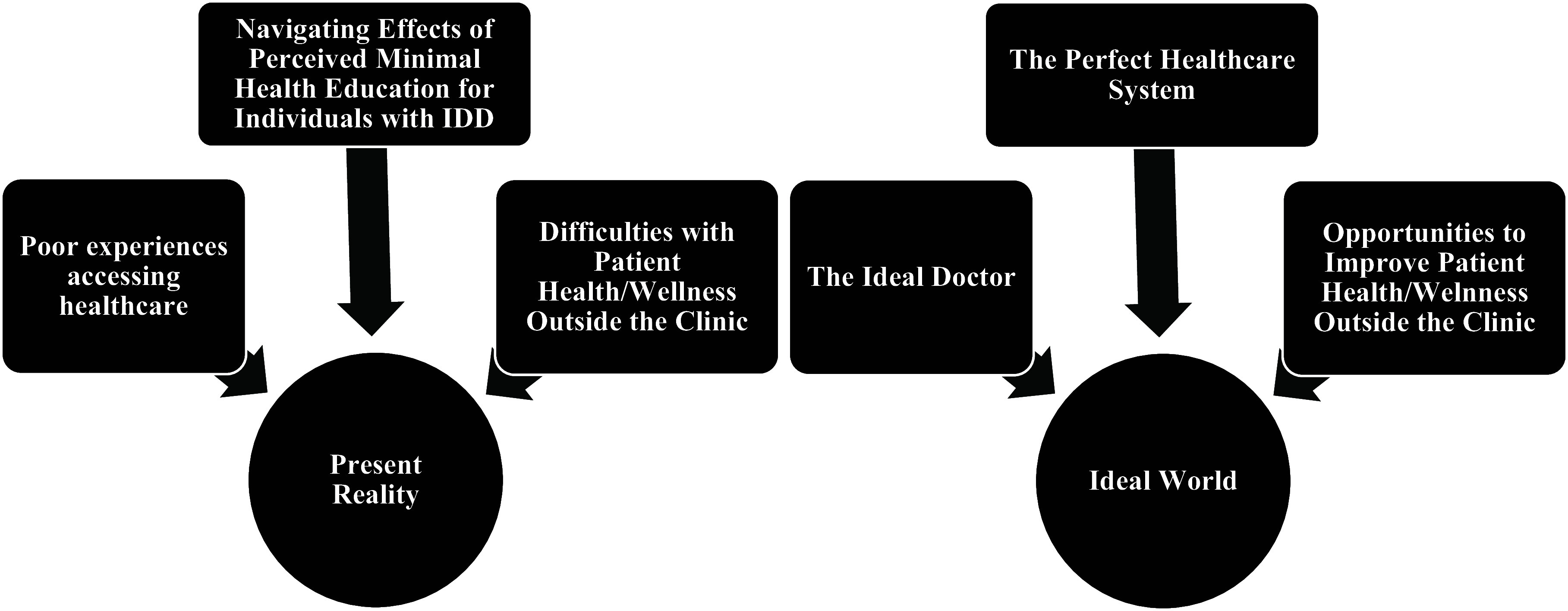
Cognitive map of focus group themes.

The questions and associated themes focused on (1) caregiver’s present reality and (2) their vision of an ideal world for preventive care services for the adults with DS for whom they provide support (Figure 2). While our sample size was small and lacked diversity, it represented a highly specific population of interest, and a smaller focus group and a longer focus group period still allowed us to achieve saturation (Guest *et al*., 2020).

#### Caregivers’ present reality

The qualitative analysis identified three subthemes from the theme of ‘Present Reality’ (Figure 2). When speaking to the interviewees about going to PCP’s physician’s office with the individual they assist, many spoke about challenges in finding a PCP provider for an adult with DC due to insurance coverage and many clinics ‘not taking new patients’. One interviewee reported: *‘We had a whole team of pulmonologists that were on my child’s care team for 2 decades. And then all of sudden they became an adult, … there was no transition at all from the pediatric team’.*

Caregivers also spoke about PCP’s lack of familiarity and knowledge of interacting with adults with DS. Some of these difficulties manifested by the PCP treating the individual as a child or talking primarily to the caregiver. One interviewee noted:

*‘It would be nice if the medical community went in with the assumption that there was more than a 2-year-old standing in front of them as a 26-year-old man. They just maybe assume that there is not a lot to him than what meets the eye’.*

Multiple caregivers also mentioned that often broader health concerns of the adult with DS were being dismissed, with PCPs focusing primarily on the DS aspect of care, or clinicians extending beyond the limits of their knowledge about this type of care.

Another subtheme revolved around maintaining beneficial preventive health practices prescribed by the PCP outside of the clinic. Many of the interviewees spoke of difficulties with maintaining a healthy diet; one interviewee said: *‘he’ll be up at 4 o’clock in the morning and eat an entire loaf of bread and I can’t watch him’.* In addition to dietary restrictions, multiple caregivers shared their struggle with establishing an exercise regimen. Some interviews stated they tried to resolve this situation by enrolling the given adult in an exercise community program which also encouraged social interaction with others who have DS.

Many of the caregivers in the focus group session agreed about the complexity of their current experiences with preventive care for the adults with DS they assist.

#### Caregivers’ ideal world

Three subthemes were identified from the theme of ‘Ideal World’ (Figure 2). The first focused on positive experiences with current physicians as well as opinions on important aspects of preventive care, including preventive services related to women’s health for female adults with DS.

Multiple caregivers emphasized the importance of finding a PCP who is willing to time with patients with DS and learn about who they are as persons.

The caregivers envisioned a PCP who is knowledgeable and willing to look for a network of specialists who can work with the person they are assisting with DS. They agreed this would reduce the burden that is felt when constantly trying to find a health services for the individuals whom they support. One interviewee provided an example of how a primary care doctor can structure this:

*‘There needs to be a checklist for the primary care physician for dentists, podiatrists, and eye doctors that they know has seen an adult with DS’.*

The second subtheme focused on how the caregivers’ desire for preventive care to be improved at a healthcare system level. Caregivers envisioned primary care clinics focused on serving individuals with DS and integrated clinics with multiple different healthcare professionals and a team approach to facilitate preventive healthcare delivery.

The third subtheme described caregivers’ desire for more access to wellness services for adults with DS. Many caregivers mentioned programs already in place through the Special Olympics organization and with other existing exercise/diet programs. The common theme regarding this topic focused on the need to make these activities as engaging as possible and to have the program include peers in their age group. One care giver offered suggestions for wellness events for adults with DS.

*‘He does not want to hear about this from parents or somebody my age, he wants somebody young. [He needs] Someone who makes them want to feel like they should keep moving’.*

#### Member checking

All seven focus group participants completed the member-checking survey. One hundred percent of the participants indicated they did not want to change or add more information to the summarized themes/subthemes of the focus group. Six out of the seven (86%) participants stated the major themes, and their subthemes matched their experiences as caregivers for adults with DS. The single participant who had marked the summary as not matching their experience agreed with the ‘ideal world’ scenario; their disagreement with the ‘present reality’ theme is provided below:

*‘I am lucky enough to have a relatively healthy son with DS, so I have not experienced the challenges of finding appropriate care’.*

Another participant elaborated further upon their agreement with the summary of the focus group and stated:

*‘Navigating the healthcare industry has been very challenging. I am hopeful we can do a better job educating younger doctors about our family members’.*

The findings or our member checking exercise confirmed the accuracy of the reviewers’ thematic analysis and illustrated the healthcare within this community.

### Survey

Eighty-one individuals completed the online survey for a response rate of 18% given the approximately 450 primary care providers (physicians and advanced practice professionals) in the Hartford HealthCare System. The demographics of the study participants are shown in Table 2.

**Table 2.** Demographics of provider survey participants

Variable	Value
**Gender,** ***n*** **(%)**	
Male	29 (35.8%)
Female	47 (58.0%)
Other/prefer not to answer	5 (6.2%)
**Race/ethnicity,** ***n*** **(%)**	
White	59 (72.8%)
Asian	7 (8.6%)
Black or African American	3 (3.7%)
Hispanic or Latino	3 (3.7%)
Two or more races	2 (2.6%)
Prefer not to answer	7 (8.6%)
**Education level,** ***n*** **(%)**	
Professional school degree	49 (60.5%)
Advanced nursing degree	16 (19.8%)
Doctoral degree	5 (6.2%)
Physician assistant degree	7 (8.6%)
Bachelor’s degree	2 (2.5%)
Master’s degree	1 (1.2%)
Other/prefer not to answer	1 (1.2%)
**Age, median (IQR)**	43.5 (36–54)
**Years in role, median (IQR)**	10 (3.5–17)

Key survey findings can be found within Table 3. The primary outcome of the survey was the resource perspective score (RPS), which assessed awareness and perspectives about resources that could aid in preventive care for adults with DS. The median RPS was 7 of a possible 23 points with an interquartile range of 6 to 9 (*N* = 68). The secondary outcome of this survey was the DSKS. The median DSKS was 11 of a possible 20 points with an interquartile range of 9 to 14 (*N* = 70). Both scores suggested less than optimal resource awareness and perspectives and knowledge about DS. A majority of the study participants indicated that they are involved in caring for patients with DS, with 66 (82%) indicating they have provided care to an adult with DS. Yet 53 (65%) reported they did not feel adequately prepared for this role, and only 22 (27%) reported they received adequate training. Less than half (49%) recalled receiving any training for caring for individuals with IDD, and only 9 (11%) received training that included DS-specific information.

**Table 3.** Provider survey outcomes and questions

*Outcomes*	Median	IQR
**Resource perspective score (** ***N*** **= 68)**	7	6–9
**Down Syndrome knowledge score (** ***N*** **= 70)**	11	9–14
***Survey questions***	**Frequency (** ***N*** **= 81)**	**Percentage (%)**
**Involved in providing care to adult with DS**		
Yes	66	81.5
No	6	7.4
Unsure	4	4.9
Did not answer	5	6.2
**Feel received adequate training to include those with DS**		
Yes	22	27.2
No	53	65.4
Did not answer	6	7.4
**Know who to contact for information to include those with DS**		
No, I do not know who to contact	56	69.2
Yes, I work within a division that has knowledgeable staff who can include those with Down Syndrome	7	8.6
Yes, I know who to contact but they are in a different division	12	14.8
Did not answer	6	7.4
**Received disability awareness training**		
Yes	40	49.4
No	36	44.4
Did not answer	5	6.1
**If yes to training, did it include DS specific information?**		
Yes	9	11.1
No	35	43.2
Did not answer or N/A	37	45.7
**Aware of AAFP yearly thyroid function test recommendation**		
Yes	26	32.1
No	43	53.1
Maybe	2	2.5
Did not answer	10	12.3

Participants were asked if they knew who to contact within their organization for support if they needed additional resources for patients with DS. Most (69%) did not know who to contact to obtain this information, 12 (15%) knew a contact from another health agency, and 7 (9%) had knowledgeable staff within their agency to provide support. Participants were also asked about their awareness of the AAFP recommendation for annual thyroid function testing for adults with DS. The majority of providers (53%) were unaware of this recommendation; 26 (23%) were aware.

In response to a forced-choice question about types of training that would be helpful for providers to provide care for adults with DS. Of the 81 participants, 46 (57%) chose ‘identifying health disparities and issues faced by individuals with DS’, 39 (48%) selected ‘engaging and supporting caregivers and persons with DS in receiving health care’, 26 (32%) chose ‘using technology to support people with DS’, and 25 (31%) chose ‘adapting the environment for persons with DS’. When asked about what resources might be useful, 46 (57%) indicated that a list of providers with training for how to care for patients with DS would be helpful and 41 (51%) said improved training content would be helpful. Finally, in response to a question about perceived barriers to care for adults with DS, 38 (47%) participants mentioned awareness barriers, 33 (41%) mentioned resource barriers, 19 (23%) mentioned communication barriers, and 12 (15%) indicated financial barriers.

## Discussion

This mixed-methods analysis found significant gaps in the adherence to preventive care guidelines for adults with DS by clinicians in Connecticut. Despite evidence-based guidelines established by the AAFP specifically for the care of adults with DS, we identified inconsistencies in the frequency and thoroughness of wellness exams and thyroid function testing between patients. For example, in rural patients in our study, thyroid testing was performed relatively more consistently than wellness visits. This could be explained both by a lack of appointment availability and fewer barriers to a ordering a thyroid test alongside a medication renewal. These findings align with previous research that identified a lack of both quantity and quality of healthcare visits (Jensen *et al.*, 2013; 2021a; 2021b).

Caregivers of adults with DS also perceived their own experiences with healthcare to be sometimes lacking in quality, mentioning issues with adequate education of clinicians on DS, ignoring aspects of general health status outside this diagnosis, and lack of support outside of the PCP office contact. We also found that clinicians felt inadequately prepared to provide primary care for patients with DS. Other studies have also found a lack of training for PCPs and insufficient resources for managing patients with disabilities (Dassah *et al*., 2018).

Further research should explore targeted interventions to address these gaps along with increased study of other DS-specific screening, including dementia, diabetes, and sleep apnea. Training programs for PCPs that emphasize the specific needs of adults with DS, along with improving access to care for adults with DS, could enhance the quality of care. Investigating the impact of these interventions on health outcomes for adults with DS provide insight into their effectiveness. Another area for future research entails exploring telehealth as a means to improve access to preventive care, especially in rural and lower-income communities where traditional healthcare access is limited. Telehealth consultations have been shown to be as effective as in-person consultations in some settings (Carrillo de Albornoz *et al.*, 2022), and it is worth investigating whether telehealth appointments would be a suitable modality to provide preventive care for adults with DS, particularly for those residing in rural areas.

## Limitations

Use of RUCA to determine home residence for adults with DS was a potential limitation. RUCA cannot identify the type of residence, for example, group home versus single-family dwelling. Additionally, RUCA does not include information on locally available services. In addition, while patients for our chart review received care from Hartford HealthCare PCPs or specialists during the study period, it is possible that patients may have transferred their care to physicians out of the network. Regarding the codes for preventive care visits, coding may have missed some visits due to differences in reimbursement as well as insurances. It is also important to consider looking at the number of PCP outpatient encounters per year. Additionally, the small sample size of the rural population may factor into some of the chart review results.

The dataset for our chart review only included patients from age 18 to 60. We chose these bounds to represent the average lifespan of this population and decreasing percentage of older individuals with DS living with a caregiver in a community setting (NCI IDD, sub-analysis for individuals with DS) and receiving care in our system’s ambulatory practices.

The sample for our focus was small, and there was limited diversity in participant demographics.

Survey participants were recruited from a single health care system, which may reduce the generalizability of the findings. The response rate of 18% may also limit generalizability, and perspectives may differ between providers who responded and those who did not, due to factors such as personal connections. In addition, the initial survey distributed via email lacked an option to input employment zip code and we were unable to compare rural and urban providers.

## Conclusion

This mixed-method study offers new insights that contribute to a better understanding of gaps in preventive care for Connecticut adults with DS. By identifying areas of improvement, it establishes a foundation for future initiatives. Targeted interventions, including physician education to enhance knowledge about preventive needs for adults with DS will be crucial to ensuring that individuals with DS receive the comprehensive, person-centered primary care required to help them lead a healthy life.

## Supporting information

Brandser et al. supplementary material Brandser et al. supplementary material

## Data Availability

All the data relevant to the retrospective chart review, the focus group, as well as the survey can be found on REDCaP, a secure research database stored through Hartford Healthcare. Data is only accessible to investigators named in the study. The data can be made available upon request and would require emailing the principal investigator, Dr. Traci Marquis-Eydman (traci.marquis-eydman@hhchealth.org).

## Supplementary material

The supplementary material for this article can be found at https://doi.org/10.1017/S1463423626101170.
