# Supplementary material for: Preventive care for adults with Down syndrome in Connecticut: a mixed-methods study
Source: Prim Health Care Res Dev. 2026 Mar 27;27:e41. doi: 10.1017/S1463423626101170 (PMC13080533; doi:10.1017/S1463423626101170)
Supplement: Brandser et al. supplementary material [file S1463423626101170sup001.docx]

**Page 1 ———————————————————————————————————**

1. What is your age?
2. What best describes your gender?
   1. Male
   2. Female
   3. Non-binary
   4. Different Identity:
   5. Prefer not to answer
3. What is your race/ethnicity?
   1. American Indian/Alaskan Native
   2. Asian
   3. Black or African American
   4. Hispanic or Latino
   5. Native Hawaiian or Other Pacific Islander
   6. White
   7. Two or more races
   8. Prefer not to say
4. What is the HIGHEST level of school you have completed or the highest degree you have received?
   1. Bachelor's degree (example: BA, AB, BS, BBA)
   2. Master's degree (example: MA, MS, MEng, MEd, MBA)
   3. Advanced Nursing degree (example: MSN, APRN, DNP)
   4. Physician Assistant degree (PA)
   5. Professional School degree (example: MD, DDS, DVM, JD)
   6. Doctoral degree (example: PhD, EdD)
   7. Prefer not to say
5. What state do you live in?
   1. Connecticut
   2. Maine
   3. Massachusetts
   4. New Hampshire
   5. New Jersey
   6. New York
   7. Pennsylvania
   8. Rhode Island
   9. Vermont
6. What health agency do you work for?
   1. *fill in the blank*
7. What division do you work for in your health agency? (Choose 1-2 divisions that best apply)
   1. Behavioral Health
   2. Children with Special Healthcare Services
   3. Family Planning
   4. Health Equity and Inclusion
   5. Mental Health
   6. Nutrition and/or Physical Activity
   7. Primary Care
   8. Sexual and Reproductive Health
   9. Specialty Care
   10. Other (please specify):
8. List the zip code of your primary workplace:
   1. *fill in the blank*
9. How many years have you worked in this role?
   1. *fill in the blank*

**Page 2 ———————————————————————————————————**

1. On a scale from 1-10 (1 no knowledge-10 extensive knowledge), how much knowledge do you have about persons with Down syndrome and their health needs?
2. If you need additional information to include people with Down syndrome in your programs/services, do you know who to contact within your health agency to receive support?
   1. Yes, I work within a division that has knowledgeable staff who can include those with Down syndrome
   2. Yes, I know who to contact but they are in a different division
   3. No, I do not know who to contact
3. Have you received any type of disability awareness training either onsite or through outside education? (Disability training is defined as training provided by employers to inform staff of how to work with and be sensitive to the special needs of persons with disabilities that will enable such persons to function and thrive in the given environment. Training includes learning about efficiently providing for the physical needs of persons with disabilities as well as dismantling stereotypes and misconceptions.)
   1. Yes
   2. No
4. If yes, did the disability training include education specifically for Down syndrome awareness?
   1. Yes
   2. No
5. Do you feel you have received adequate training from your health agency to include those with Down syndrome in your programs and services?
   1. Yes
   2. No
   3. Depends (explain):
6. If provided knowledge, skills, and technical assistance for accommodation, would you include people with Down syndrome in your programs and services?
   1. Extremely unlikely
   2. Somewhat unlikely
   3. Somewhat likely
   4. Extremely likely
7. Have you or your healthcare team ever been involved in providing care for an adult with Down Syndrome?
   1. Yes
   2. No
   3. Unsure

**Page 3 ———————————————————————————————————**

1. What types of **training** would be helpful? (Select up to 3 options)
   1. Ableism awareness training
   2. Adapting the environment for persons with Down syndrome
   3. Engaging and supporting caregivers and persons with Down syndrome in their health care
   4. Identifying health disparities and issues that people with Down syndrome face
   5. Implementing Universal Design for Learning
   6. Inclusive language training (including person-first terminology)
   7. Tailoring Down syndrome-specific training for healthcare professionals
   8. Provisions of the Americans with Disabilities Act (ADA)
   9. Using technology to support people with Down syndrome
   10. Other (please specify):
2. What types of **resources** would be helpful? (Select up to 3 options)
   1. Infographics on health for those with Down syndrome
   2. Issue briefs on policy issues affecting the health of those with Down syndrome
   3. Training content on a learning portal
   4. Live-facilitated training session(s)
   5. Up-to-date information and newsletters via email
   6. List of health providers that are trained on caring for people with Down syndrome
   7. Opportunities to volunteer at events with people with Down syndrome
   8. Other (please specify):
3. Are people with Down syndrome and/or their caregivers engaged in the planning and delivery of services and programs?
   1. Yes, all the time
   2. Some of the time
   3. No, not at all
4. Which barriers prevent your health agency from including people with Down syndrome in programs and policies? (Select all that apply)
   1. Attitudinal barriers (e.g., stereotyping, stigma, prejudice, and discrimination)
   2. Awareness barriers (e.g., lack of knowledge and experience to serve Down syndrome population)
   3. Communication barriers (e.g., inadequate staff communication and coordination, lack of Down syndrome-friendly language and messaging)
   4. Financial barriers (e.g., lack of sufficient funding to provide services and/or collect data)
   5. Physical barriers (e.g., lack of mobility-friendly infrastructure and transportation)
   6. Resource barriers (e.g., insufficient time and staff available needed to serve the community)
   7. Organizational competing interests
   8. Other (please specify):

**Page 4 ———————————————————————————————————**

1. Do you know the percentage of people in your service area that have ID?
   1. Yes
   2. No
   3. Maybe (explain):
2. Do you know the percentage of people in your service area that have Down syndrome?
   1. Yes
   2. No
   3. Maybe (explain):
3. Are you aware of the American Academy of Family Physicians’ recommendation to order a thyroid function test every year at the annual wellness visit?
   1. Yes
   2. No
   3. Maybe (explain):
4. Is this recommendation followed within your health department?
   1. Yes
   2. No
   3. Unsure
   4. N/A
5. If a person with IDD does not achieve their annual wellness goals, who or what do you think is most likely responsible?
   1. The person/patient’s family or guardian
   2. The person/patient
   3. The healthcare system
   4. The primary care provider
   5. The group home (if applicable)
   6. Multifactorial
   7. No one’s fault or responsibility
